# Using provocative design to foster electronic informed consent innovation

**DOI:** 10.1186/s12911-022-02039-6

**Published:** 2022-11-17

**Authors:** Evelien De Sutter, Stef Verreydt, Koen Yskout, David Geerts, Pascal Borry, An Outtier, Marc Ferrante, Corinne Vandermeulen, Nele Vanmechelen, Bart Van der Schueren, Isabelle Huys

**Affiliations:** 1 Clinical Pharmacology and Pharmacotherapy, Department of Pharmaceutical and Pharmacological Sciences, KU Leuven, Leuven, Belgium; 2 Distributed and Secure Software (DistriNet), Department of Computer Science, KU Leuven, Leuven, Belgium; 3 KU Leuven Digital Society Institute, KU Leuven, Leuven, Belgium; 4 Centre for Biomedical Ethics and Law, Department of Public Health and Primary Care, KU Leuven, Leuven, Belgium; 5 Department of Gastroenterology and Hepatology, University Hospitals Leuven, KU Leuven, Leuven, Belgium; 6 Leuven University Vaccinology Centre, Department of Public Health and Primary Care, KU Leuven, Leuven, Belgium; 7 Department of Endocrinology, University Hospitals Leuven, KU Leuven, Leuven, Belgium; 8 Clinical and Experimental Endocrinology, Department of Chronic Diseases and Metabolism, KU Leuven, Leuven, Belgium

**Keywords:** Qualitative research, Provotypes, Ethics, Digital consent, Human-centered design, User interface, Clinical study

## Abstract

**Background:**

The development of technological applications in clinical research, such as electronic informed consent (eIC), is on the rise. The involvement of end users throughout the design process of eIC is of utmost importance to improve the current informed consent process.

**Methods:**

Using a provocative design, we conducted interviews with 30 clinical research participants. Provotypes were used as a starting base to discuss various aspects relevant to eIC. By providing a medium to encourage divergent thinking, participants’ views and concerns were solicited. Thematic analysis was undertaken using NVivo.

**Results:**

The majority of participants placed trust in the principal investigator or the hospital to perform the role of eIC hosting party. Differing opinions were reported on the amount of information required related to stakeholders’ access to an eIC system, and thus, to participants’ personal data, to enable trust in an eIC system. Nevertheless, this study indicates a general willingness of participants to share personal data with physicians and pharmaceutical companies on an international level, and to receive requests for new research studies via an eIC system. Participants suggested to tailor an eIC system based upon their preferences, for example, regarding whom they want to share their personal data with. Moreover, they expressed a desire to choose how they can contact the research team, and to indicate which study-related information they would like to receive electronically. In addition, positive opinions were voiced on the integration of a test to assess participants’ understanding before providing their eIC.

**Conclusions:**

Following a research through design approach, insights have been generated which inform the design of eIC. Provotypes were designed to help participants think beyond what is familiar to them. Study findings revealed that not all situations were perceived as provocative, because of participants’ motivation to advance scientific research and the trust they place in the research team. Nevertheless, the use of provocative design resulted in additional insights, generated by clinical research participants, which could be considered in the further design of eIC.

**Supplementary Information:**

The online version contains supplementary material available at 10.1186/s12911-022-02039-6.

## Background

Digital health, such as health information technology or telemedicine, is becoming increasingly part of our healthcare system and holds huge promise for improving patient-centered care [1–4]. If purposefully designed, digital health applications can promote the quality of health services and ultimately, citizens’ welfare [5]. In the context of clinical research, substantial interest has been paid to electronic informed consent (eIC). For example, the European Medicines Agency issued guidance during the COVID-19 pandemic, specifically addressing the use of eIC to inform participants and obtain their consent [6]. eIC holds great potential to encourage research participant engagement in the informed consent (IC) process [7]. Over time, IC forms have become lengthy documents, containing in-depth legal and scientific information, which are often complex to read and understand by research participants [8, 9]. According to various stakeholders involved in clinical research, eIC could allow a personalized approach and could establish ongoing communication between participants and the research team [7, 10]. For example, participants could be able to indicate for which reasons they would like to be recontacted via an eIC system or a test could be implemented to assess their comprehension [10].

End users have been traditionally considered passive recipients of digital health [11]. As a result, digital technologies were seldom developed by using human-centered design principles, which is considered a key factor to achieve successful implementation [12–14]. However, during the past years, the interest has grown to involve end users in the design of digital technologies [12, 15]. Collaborating with end users aims to facilitate the development of technologies that serve the end users’ needs [16]. With regard to clinical research, it is essential that clinical research participants should play a key role in the design of an eIC system [17].

To inform the design of an eIC system, provocative design can be used to elicit feedback from end users. Provocative design intentionally provokes end users to generate detailed reactions by triggering their creativity and bringing them out of their comfort zone [18, 19]. As a result, divergent ideas related to the design of an eIC system may be generated [20]. Various types of provocation are described in literature. For example, aesthetic provocation refers to the visual characteristics of the design, functional provocation challenges the ways of interacting with the design, and conceptual provocation is related to the concepts the design tries to challenge [21, 22]. The conduct of provocative design is facilitated by the use of provocative prototypes, also referred to as provotypes, which are considered design artefacts [23]. Provotypes visualize issues or perspectives considerably further than spoken words, and therefore, aim to overcome barriers related to unlocking end users’ thinking [23]. To define opportunities for a user-friendly eIC system, we aim to investigate how provotypes might trigger and shape the views of clinical research participants on different aspects relevant to the design of eIC. Moreover, we aim to gain insights into the importance participants give to particular ethical and legal requirements. The opinions provided during this study may inform the design of a participant-centric eIC system in clinical research.

## Methods

### Design

This manuscript builds on results from previous research related to eIC [7, 10]. Previous research has shown that research participants stress the importance of trust in the authenticity of eIC, which goes hand in hand with the party that should ideally host an eIC system [7]. Therefore, participants’ views regarding the hosting party were investigated. Additionally, the amount of information that participants would like to receive regarding stakeholders’ access to an eIC system and, thus, to participants’ personal data, were also addressed. The first and second provotypes aimed to deepen understandings on what participants value in the design of an eIC system that would hold their trust. Other challenges for the design of eIC lie in the personalization and long-term interaction aspects. Therefore, our provotypes challenged participants on (1) being searchable for future research studies, (2) being electronically informed about study-related information, (3) how to contact the principal investigator (PI) during the course of the study, (4) providing reconsent, (5) the responsibility they might have when indicating their preferences, and (6) the implementation of questions to assess their understanding of study-related information. In total, eight provotypes were used in this study that may conceptually or functionally provoke participants and touch upon the themes of trust, personalization or the long-term interaction. Provotypes, created with the software program Figma, presented various situations, ranging from provocative (i.e., intended for critical reflection) towards prototypical (i.e., intended to test a certain design direction). A detailed description and visualization of the provotypes can be found in Table 1 and Additional file 1, respectively. These provotypes were used during interviews with participants who have taken part in a clinical trial. Participants interacted with these provotypes as if it would concern a real eIC system. For example, it was pretended that participants needed to log-in in order to access the different situations. During each interview, the eight provotypes, including the various situations, were shown in the same order, starting from the most provocative design and ending with the most prototypical design. After each situation, the participant’s opinion was asked. At the end of the interview, the participant’s demographic data (i.e., age and highest education level) were collected. Moreover, it was clearly explained that some situations might not be correct from an ethical and legal point of view in order to avoid confusion.

**Table 1:** Description of the provotypes presented during the interviews

*Provotype 1: Hosting an eIC system*
Participants were shown four different interfaces. Each interface mentioned that a certain party is responsible for hosting an eIC system, and thus, for correctly storing participants’ personal data and guaranteeing their privacy. Hosting parties included (1) a pharmaceutical company, (2) the Belgian government, (3) the PI, and (4) the hospital that has been conducting the clinical trial in which the participant has taken part.
*Provotype 2: Amount of information about stakeholders’ access to an eIC system*
Participants were shown four different interfaces. On each interface, it was mentioned that an eIC system stores participants’ personal data such as their name, address, date of birth, and medication history. Additionally, interfaces included the following messages: (1) “We cannot tell you who has access to your personal data.”, (2) “Only persons with legitimate rights have access to your personal data.”, (3) “The Belgian government has access to your personal data when conducting inspections. The government will conduct inspections if deemed necessary, for example if there is a complaint related to the study. The PI has access to your personal data when consulting your electronic health record. The evaluating ethics committee does not have access to your personal data. The task of the ethics committee is to protect the rights of the participants who take part in a clinical study. They verify if your rights are respected. The sponsor of this study does not have access to your personal data. The sponsor is responsible for the preparation of the research protocol, taking an appropriate insurance, receiving approval for conducting the study of the relevant authorities/ethics committees, and registering the study in a publicly available register.”, and (4) “The Belgian government and the PI have access to your personal data.”.
*Provotype 3: Being searchable for future research studies*
Participants were shown three different interfaces. On each interface, it was mentioned that an eIC system stores participants’ personal data such as their name, address, date of birth, and medication history. Participants were informed that their data will be shared with (1) physicians all over the world, (2) a specific pharmaceutical company, and (3) physicians of the hospital that has been conducting the clinical trial in which the participant has taken part, and that they could contact them when they need participants for a new research study. All interfaces indicated that participants are able to indicate for which particular type of research (e.g., for research in a particular health condition) they would like to be contacted.
*Provotype 4: Being electronically informed about study-related information*
Participants were shown one interface on which five pop-up messages appeared one after another. These messages included the following information: “The recruitment of this study has been finished. The study will start soon.” or “New results of the study in which you are taking part are available. Please consult these results.” or “There is a new IC version available. Please consult this new version.”. Some messages appeared twice.
*Provotype 5: Contacting the PI during the course of the study*
Participants were shown three different interfaces. Interfaces included the following messages: (1) “If you have questions about this clinical study, you can only contact the chatbot. The chatbot is a software program that will help you further.”, (2) “If you have questions about this clinical study, you can only contact the PI via the chat.”, and (3) “If you have questions about this clinical study, you can only set-up a video consultation with the PI”.
*Provotype 6: Providing reconsent*
Participants were shown three different interfaces. Interfaces included the following messages: (1) “There is a new IC version available which you can consult. You will automatically agree to this version and do not need to take any action.”, (2) “There is a new IC version available which you can consult. The changes are highlighted. If you agree, please sign this version electronically.”, and (3) “There is a new IC version available which you can consult. The changes are highlighted and additional information is provided on why these changes were necessary. If you agree, please sign this version electronically.”.
*Provotype 7: Participants’ responsibility when indicating their preferences*
Participants were shown two interfaces. On the first interface, it was mentioned that participants could be informed about the further course of the clinical trial via an eIC system, and that they could indicate their preferences regarding the type of information they would like to receive. The second interface included various options participants could choose. Options included: (1) the status of the study, (2) final study results, (3) preliminary study results, (4) results of additional investigations (e.g., blood sampling), and (5) a new IC version.
*Provotype 8: Implementation of questions to assess participants’ level of understanding*
Participants were shown four different interfaces. Interfaces included the following messages: (1) “Please answer the questions below. You must answer all questions correctly before you can sign the IC. If you need more information to answer a question, please consult the PI. This system will not provide any feedback or additional information on your answers, except whether all questions were answered correctly. If you give an incorrect answer to one or more of the questions, you will be excluded from the study. You get one single attempt.”, (2) “Please answer the questions below. You must answer all questions correctly before you can sign the IC. If you need more information to answer a question, consult the PI. This system will not provide any feedback or additional information on your answers, except whether all questions were answered correctly. You can retry as many times as you want.”, (3) “Please answer the questions below. You must answer all questions correctly before you can sign the IC. If you need more information to answer a question, you can click the ‘Tell me more’ button. For any questions that are answered incorrectly, you will receive additional explanation. You can retry as many times as you want.”, and (4) “Please answer the questions below if you want to get insights in how well you understand the study in which you would like to take part. It is NOT necessary to answer all questions correctly before you can sign the IC. If you need more information to answer a question, you can click the ‘Tell me more’ button. You can retry as many times as you want, or click ‘skip’ to sign immediately.”.

### Recruitment

Recruitment was done in Leuven, Belgium, by the Department of Gastroenterology and Hepatology, and the Department of Endocrinology at the University Hospitals Leuven, as well as the KU Leuven Vaccinology Center (LUVAC), through purposive sampling. Additionally, the researcher herself (EDS) recruited subjects at the Department of Endocrinology at the University Hospitals Leuven. Participants were eligible for inclusion if they were over 18 years old, were Dutch-speaking, and had experience with taking part in an observational or an interventional clinical trial. Participants with prior trial experience were selected so they could better empathize with the situations we presented them with. The recruiting parties provided an invitation, by email or in person, to potential participants. The invitation contained information on why they were invited to participate, the objectives and course of the interview study, the evaluation of the study by the Ethics Committee Research UZ/KU Leuven, the compensation participants would receive after participation, and the contact details of the researcher. If interested, participants could contact the researcher by email or phone. Written IC was obtained from each participant before the start of the interview. Afterwards, they received a gift voucher of 25 euro. Recruitment ceased once data saturation was achieved.

### Data collection and analysis

Interviews were conducted either face-to-face or by using Microsoft Teams, according to the preference of the participant, between July and September 2021. All interviews were conducted by the same researcher (EDS). When interviews were conducted remotely, the participant was given control of the researcher’s screen in order to interact with the provotypes. Interviews lasted between 30 and 60 min, were conducted in Dutch, and were digitally audio-recorded. Thematic analysis was performed in accordance with the various stages of the framework method, described by Gale et al. [24]. A description of each stage is presented in Table 2. Dutch quotes were translated to English upon inclusion in this manuscript.

**Table 2:** Description of the framework method stages

Stages	Additional explanation
1. Transcription	Pseudonymized audio-recordings were transcribed a verbatim by a third-party
2. Familiarization with the interview	The researcher (EDS) thoroughly read the transcripts, made reflective notes, and relistened to the audio-recordings, if necessary
3. Coding	The researcher (EDS) coded 2 transcripts via a hybrid deductive and inductive approach. The provotypes presented during the interviews informed the creation of deductive codes while inductive codes were created based upon observed patterns
4. Developing a working analytical framework	Codes sharing similarities in content were grouped into categories, creating a working analytical framework
5. Applying the analytical framework	The researcher (EDS) applied the working analytical framework, also referred to as the coding tree (Additional file 2), to the other transcripts by using NVivo software
6. Charting data into the framework matrix	The data were charted into the framework matrix. Microsoft Excel was used to summarize the data from each code and transcript
7. Interpreting the data	The data were interpreted to identify similarities and differences between the data

## Results

In total, 30 interviews using a provocative design were conducted with participants who have taken part in various clinical trials: (1) an interventional vaccine trial (n = 12), (2) an interventional trial in the field of inflammatory bowel disease (n = 10), and (3) an observational trial in the field of diabetes (n = 8). Participants’ characteristics are shown in Table 3. Results of the interviews were reported for each provotype related to trust, long-term interaction, and personalization.

**Table 3:** Participant demographics

Characteristics	Interviewees (n = 30)	
	N	%
*Age range (years)*		
20–39	8	27
40–59	7	23
60–83	15	50
*Sex*		
Male	17	57
Female	13	43
*Education*		
High school	10	33
Bachelor’s degree	15	50
Master’s degree	5	17

### Trust

#### Hosting an electronic informed consent system

Generally, participants expressed confidence in a pharmaceutical company, the Belgian government, the PI, and the hospital where the clinical trial is conducted to perform the role of hosting party. It was argued that each party must comply with the applicable legislation regarding data protection. There were participants who expressed preferences for the PI and the hospital to host an eIC system. However, few participants indicated that they participate in a trial to advance scientific research, and that they do not take the hosting party in consideration. Moreover, it was believed that receiving information about the hosting party could foster clinical trial transparency. To this end, it is recommended to provide specific information about the department—within the Belgian government, the hospital or the pharmaceutical company—that would be responsible for hosting the system.

Initially, several participants had reservations about a pharmaceutical company as hosting party, due to their profit-seeking behavior. Nevertheless, these participants highlighted that the efforts of pharmaceutical companies to speed up the process of bringing COVID-19 therapeutics to the market had a positive impact on the trust they have in the industry. The Belgian government was considered the most neutral party to host an eIC system. Participants indicated that they are already familiar with the use of applications hosted by the government, for example, to fulfil tax obligations or to receive a COVID-19 vaccination certificate. Therefore, they raised the point that they can trust the government. Nevertheless, it was raised that the government as hosting party of an eIC system sounded strange at first. According to the participants, it would be reasonable that the hospital would host an eIC system. Participants’ medical information is documented in electronic health records that are stored in the hospital. Therefore, participants believe that the hospital has sufficient safeguards in place to adequately protect their data. Finally, almost all participants expressed confidence in the PI of the clinical trial in which they have taken part.

“Because I have developed a relationship of trust with the PI, this person can be put in control of my personal data” (P19).

Nevertheless, one participant asserted that it would not be feasible for one person to host an eIC system. Moreover, it is more likely that the PI would, due to the lack of IT skills, outsource the hosting to another department or organization.

#### Level of detail on access information

Generally, participants did not agree with the first interface, which states that it is not possible to provide information on access of stakeholders to the participants’ personal data. It was highlighted that this situation is not acceptable and does not result in an eIC system that would hold the participants’ trust. Moreover, questions arose regarding the reason why it is not possible to provide the necessary information. Several participants argued that additional information about stakeholders’ access was preferred, whereas only a few other participants were confident that only the necessary persons would access their personal data. Participants were more positive towards the second interface, specifying that only persons with legitimate rights have access to participants’ personal data. Nevertheless, participants stated that they must be able to read more specific information about the persons with legitimate rights. Therefore, there were participants who expressed preferences for the third or fourth interface that includes minimal and detailed information about stakeholders’ access to participants’ personal data, respectively. Some participants noted that, because of the relationship of trust that is developed with the clinical research team, the minimal information is sufficient, whereas others argued that detailed information is necessary to enable trust in an eIC system. Therefore, some participants advised to offer all necessary information.

“Although the detailed information is time-consuming to read and process, it must be made available to all participants” (P29).

Moreover, it was advised to structure the information, for example by using bullet points, and to specify the department of the Belgian government that could access participants’ personal data.

### Long-term interaction

#### Being searchable

This provotype sought to investigate participants’ preferences for sharing personal data and being contacted for new research studies. There were participants who wanted to share their personal data with physicians all over the world, the pharmaceutical industry as well as physicians of the hospital that has been conducting the study in which the participant has taken part. Participants tended to consider being contacted by these parties for new research studies as desirable. Moreover, it was considered valuable that participants are given the choice to indicate for which type of studies they can be contacted. Various interviewees saw this as an opportunity to participate in a study that could further the research field of the condition that affects them.

“I would immediately volunteer to participate in a study related to diabetes to advance research in this health domain” (P26).

However, some participants raised the concern that they do not want to be overloaded with requests, and therefore they stressed the need to set preferences for being notified. For example, participants could be offered the possibility to set a limit on the notification frequency or to indicate how they would like to be contacted. Other considerations on this matter are shown in Table 4.

**Table 4:** Participants’ considerations on being searchable

When personal data is shared with physicians all over the world or with a pharmaceutical company, larger clinical studies can be conducted to advance scientific research
Concerns were raised about the differing privacy rules at a global level
When personal information is shared only with physicians of the hospital that has been conducting the study in which the participant has taken part, possibilities are limited from a scientific point of view
A desire was expressed to be able to tailor preferences about whom participants want to share their personal data with
Trust in a new research study, conducted by foreign physicians or a pharmaceutical company, may be positively impacted by discussing the study with the participant’s treating physician
Specific information must be provided on the name of the pharmaceutical company

#### Being alerted about new information

Some participants indicated that the pop-up messages, describing that new study-related information is available, help to capture their attention. It was suggested that these messages could include a link that automatically redirects participants to more detailed information. Nevertheless, showing multiple pop-up messages one after another was considered annoying and overwhelming. In order to avoid a disorganized overview of new information, participants advised to use an inbox-like format rather than pop-up messages.

The majority of participants asserted that they would forget to undertake the action of logging in to an eIC system to verify if new study-related information has been made available. It was believed that a notification, such as an email, can be sent to participants, alerting them that new information can be accessed on an eIC system. As a result, participants are given the choice on whether or not to consult the information. Nevertheless, participants prefer some control over the type and frequency of notifications they receive.

Moreover, participants raised the issue that they are already using a health-related application that sends notifications about new information in their medical dossier or medical appointments. Therefore, they indicated that it would be valuable to integrate the clinical study-related information, and thus, an eIC system, into established systems.

### Contact with the principal investigator

Participants indicated that it could be possible to obtain support through a chatbot. It was considered a good starting point to inform participants on a continuous basis. Participants mentioned that a chatbot could respond to frequently asked questions related to the conduct of clinical studies. Nevertheless, the personal contact between the participants and the research team was valued highly. Therefore, participants raised that if they have specific questions that require further explanation, they must have the possibility to contact the research team.

“If you engage in taking part in a study, you do not want to receive automated responses that do not address your question” (P2).

Participants agreed that the utilization of a chat feature can support the direct interaction with the PI. Chat integration allows the participants to discuss questions with a trustworthy person who has medical expertise. Participants indicated that not only the PI, but also another member of the research team, can answer their questions.

“The research team is informed about my medical background and is able to provide specific study-related information” (P20).

Nevertheless, participants required clarification on when it would be possible to chat with the research team. Moreover, some participants mentioned that they are unaccustomed to the interaction with others over technology, and therefore, prefer a face-to-face conversation.

Video consultations were considered as real-time interactions between the participants and the PI. It was raised that a video consultation enables participants to capture the body language and facial expression of the PI. Similar to the chat function, participants required clarification on when a video consultation could be scheduled. One person highlighted that, when an appointment must be booked, additional efforts are required to ask for clarification. Additionally, some participants were concerned that conducting video consultations would result in an increased workload for the PI.

Some participants stated that if only one option is provided to contact the PI or the research team, it would adversely affect participants’ willingness to take part in a particular study. Therefore, they advised to offer several options. Generally, participants indicated that their preferences depend on the importance of their questions and their familiarity with technology. If it concerns a non-urgent question, it can be asked via chat, email or on the participant’s next medical visit. However, in emergency situations where a participant has developed acute symptoms during the course of the study, there must be the possibility to call the PI or the research team.

### Signing new versions of electronic informed consent

It was raised that automatically agreeing to a new eIC version, as mentioned in the first interface, is a convenient option. Participants expressed confidence in the research team, and therefore, believed that it would concern ethically sound modifications. Nevertheless, several participants voiced the opinion that it is not acceptable to automatically agree to a new eIC version and questioned the need to read the new version in case of automated approval.

“Why would I consult the new eIC version if it has been automatically accepted?” (P12).

These participants emphasized the importance of indicating their agreement through a clear affirmative action, for example by ticking a box. Moreover, they advised to transparently communicate the modifications of the new eIC version. For this reason, they agreed with the second interface, stating that the changes of the new eIC version are indicated. Because it often concerns lengthy IC forms, participants raised that they should be able to immediately notice the changes.

The third interface, mentioning that additional information on the modifications is provided, was appreciated by the majority of participants. Some participants raised that they still have the option on whether or not they access the additional information.

“You could have the choice to access the additional information. It would also be interesting for the research team to monitor how many participants have read that information” (P25).

Nevertheless, it was considered burdensome for the research team. Therefore, they advised to provide only additional information if the research team deems it necessary. Finally, participants highlighted that it is important to mention that if they would have questions about the new eIC version, they have the possibility to contact the research team.

### Personalization

#### Responsibility

Generally, participants did not feel personal responsibility to express their preferences regarding the type of study-related information they would like to receive. They reported that their involvement is encouraged by indicating which information they would like to be informed about, and mentioned that the added value of eIC is the access to additional study-related information.

“Because participants may have different interests, it is valuable that it can be indicated which information someone wishes to receive” (P4).

Almost all participants were interested in receiving study results. More concretely, they were willing to receive the final results and considered the preliminary results as redundant. However, concerns were raised related to the understandability of the results. Differing perceptions were reported on the willingness to receive information on the status of the study or additional investigations. For the latter, one participant mentioned that it would be useful to be informed in case of deviating results only. Finally, the majority of participants wanted to receive new IC versions electronically. They felt that receiving the information digitally could promote environmental sustainability.

#### Questions to assess participants’ level of understanding

Generally, participants were positive towards the implementation of a test into the eIC process to assess their understanding of study-related information. These questions require them to reflect on what the study entails and to consult the study team if additional clarification would be required. Participants emphasized the importance of understanding the study conduct and implications before being enrolled in the study. They believed that the implementation of questions can depend on the type of study. For example, a test was considered more valuable for interventional rather than for observational studies because of the higher safety risk, related to the intervention, posed to study participants. Additionally, participants stressed the need to mention the aim of the questions: creating awareness of study-related information. Moreover, they highlighted that they must be informed, in advance, that they need to complete questions and when they have to do so.

Participants widely disagreed with the first interface, indicating that they have only one single attempt to answer all questions correctly. According to the participants, unintentional errors are often likely be part of a question session, depending on the type and difficulty of questions. Therefore, it was raised that having one attempt would adversely affect recruitment. Participants also indicated that it is not acceptable to exclude patients with a specific disease who consider a clinical trial as an opportunity to improve their health condition. The first interface also described that participants could consult the PI to receive additional information to answer a question. However, some participants thought that it is not always feasible to reach the PI and acknowledged that they do not want to disturb the PI.

The second interface offered participants the possibility to retry as many times as they want to answer all questions correctly. Nevertheless, participants advised to set a limit on the number of attempts in order to avoid gambling.

“If you have not understood the information, it is useless to answer the questions multiple times” (P14).

Moreover, it was suggested to receive additional explanation when a question would be answered incorrectly, as presented on the third interface. Additionally, this interface enables participants to click the ‘Tell me more’ button before answering the question, which participants considered valuable. Nevertheless, if the additional information would not suffice, participants want to have the possibility to contact the research team.

When participants were shown the fourth interface, stating that it is not necessary to answer all questions correctly before signing the IC or that questions can be skipped, they believed that in some situations the questions would not be completed. For example, if it would concern a patient who lacks energy or if a long and time-consuming questionnaire needs to be filled out. Nevertheless, participants were of the opinion that, if the questions would be skipped, clinical research participants may not be aware of their responsibility when taking part in a study.

## Discussion

User involvement has become a widely accepted principle in the development of user-friendly technologies and is considered crucial to improve the quality of digital health [15]. However, limited literature exists on human-centered design to inform the creation of an eIC user interface [7]. Nevertheless, the importance of engaging clinical research participants in the design of an eIC interface cannot be understated. They are in a unique position to share their experiences about taking part in a clinical study. Recognizing the need to involve clinical research participants in the design process of a participant-centric eIC system is what resulted in the study described in this manuscript. Our study confirms that participants can reflect on several situations in order to explore future eIC design possibilities. Provotypes, putting forward more or less provocative situations relevant to the design of eIC, were a useful strategy to engage participants in an open discussion. The following key considerations for the design of an eIC system can be made based on our results.

### Trust

The majority of participants showed a preference for the PI or the hospital to perform the role of hosting party, as confidence was expressed that they handle participants’ personal data properly. Similarly, another qualitative study involving various clinical research stakeholders found that the PI, a regulatory body or a trusted center, controlled by a regulatory body, could be responsible for hosting an eIC system [10]. A scoping review has shown that trust in digital health depends on a variety of factors. For example, the reputation of the service provider can act as an enabler or impediment to have trust in technologies [25]. Several participants highlighted that a pharmaceutical company is motivated by profit and therefore, expressed more trust in public institutions, which is in line with the available literature. A cross-sectional study conducted among patients showed that their willingness to participate in a clinical study depends on the institution that is sponsoring the study. These patients indicated to be more willing to participate in a study sponsored by a public institution rather than a pharmaceutical company [26].

According to Beauchamp and Childress, respect for a person’s autonomy is one of the principles that lies at the core of biomedical ethics [27]. Nevertheless, study findings suggest that some participants felt indifferent towards the impairment of their autonomy once a relationship of trust has been developed with a specific professional or institution. For example, some participants agreed to not being able to make an autonomous decision about whether or not to accept a new eIC version because of the trust they have in the research team. Within health-related research, trust is a prominent factor between the study participant and the research team [28]. For this reason, making a technological application, such as eIC, trustworthy is critical to support this participant-research team relationship that involves trust.

### Control and transparency

The provocative interfaces used in our study aimed to openly explore various themes. Participants did not always agree with statements mentioned on these interfaces, for example, related to automatically agreeing to a new eIC version or being searchable by specific parties. Therefore, it seems that some participants want to be offered a certain level of control to take decisions, such as indicating with whom they want to share their data with and for which type of studies. The concepts of control and transparency are inextricably linked. Transparent information needs to be conveyed to participants to address potential concerns they may have and is considered important to garner a justified trust in eIC [29, 30]. When purposefully designed, eIC affords the opportunity to establish a transparent information exchange: it may enable participants to control their decisions, based on their wishes, and to receive understandable information over the course of the study.

### Flexibility

Generally, participants’ preferences differed regarding the type and amount of study-related information they would like to access. Additionally, participants wanted to have various options to contact the research team. These divergent views require the need for the design of a flexible eIC interface that can be tailored to the participants’ wishes. In doing so, eIC may overcome the static character of a paper-based IC form, which is one of its stumble blocks [10]. Existing literature shows that the information participants consider important for their decision to participate in a clinical study differs [31]. A flexible approach can accommodate these differences, for example, by offering a layered eIC model. The first and second layer can convey essential and more detailed information, respectively [10]. Moreover, by enabling participants to indicate which aspects of a clinical study they would like to be informed about, they may have more control over their involvement in this study [10, 32].

### Informed decision

Each participant may have differing preferences, values, and goals which can impact the process of making an informed decision on enrolling in a clinical study. A flexible eIC interface can aid participants in accessing study-related information based on their needs and interests. Moreover, to ensure that participants understand the information, it has been suggested to integrate a test in the eIC interface to assess their comprehension [7, 10]. Generally, participants involved in this qualitative study were positive towards the implementation of a test. However, it is recommended to design an eIC interface providing access to additional information and answer feedback during and after completion of the test, respectively. Wilbanks et al. designed an eIC interface in which a test was implemented [33]. In initial studies, participants needed to answer all questions correctly whereas in subsequent studies, participants could also enroll when one or more questions were incorrectly answered [34, 35]. Wilbanks described that the latter approach is mainly appropriate for lower-risk observational studies [35]. Similarly, participants involved in our study voiced that the importance of a test depends on the type of study.

### Strengths and limitations

eIC has received little attention from a user-centered design perspective. Therefore, this study’s main strength lies in engaging clinical research participants, as one of the important end users of an eIC system, in the design of an eIC interface. Provocative interviews are qualitative in nature and, therefore, provide subjective evidence that may not be generalizable. Nevertheless, our study design aimed to involve participants who have taken part in diverse types of clinical studies to reduce specificity. Additionally, we noticed that participants’ ages ranged from 20 to 83 years, resulting in a heterogeneous sample. Nevertheless, it should be mentioned that the recruitment of participants was conducted in Belgium only. All interviews were conducted by the same researcher, reducing the variability between interviews. Participants’ experiences with taking part in a clinical study, and more specifically with the IC process, was highly relevant to inform the design of an eIC interface. However, it is important to recognize that they provided views in a hypothetical setting, which may not reflect their decisions in more naturalized circumstances. In addition, it should be noted that the coding of the data was performed by one researcher. Although no independent coder verified the code assignment, the use of the other stages of the framework method and the availability of existing literature to inform the coding process minimized subjective interpretation of the data [10, 24].

## Conclusion

To assist responsible implementation of eIC in clinical research, it is important to put study participants’ desires and concerns at the forefront. This manuscript describes the involvement of study participants in the design process of eIC. Through provocative design, participants are triggered to creatively reflect on situations related to trust, personalization, and the long-term interaction. Findings point to the importance of trust, control & transparency, flexibility when designing eIC systems, and making an informed decision. These findings will contribute to the design of a participant-centered eIC system.

## Supplementary Information

**Additional file 1: **Visualization of provotype 5: contacting the principal investigator during the course of the study.

**Additional file 2: **Coding tree.

## Data Availability

The datasets generated and/or analyzed during the current study are not publicly available due to confidentiality requirements but are available from the corresponding author on reasonable request.

## Abbreviations

eIC: Electronic informed consent
IC: Informed consent
PI: Principal investigator
