# Supplementary material for: Using provocative design to foster electronic informed consent innovation
Source: BMC Med Inform Decis Mak. 2022 Nov 17;22:296. doi: 10.1186/s12911-022-02039-6 (PMC9669523; doi:10.1186/s12911-022-02039-6)

**Using provocative design to foster electronic informed consent innovation**

Evelien De Sutter^1^, Stef Verreydt^2^, Koen Yskout^2^, David Geerts^3^, Pascal Borry^4^, An Outtier^5^, Marc Ferrante^5^, Corinne Vandermeulen^6^, Nele Vanmechelen^7^, Bart Van der Schueren^7,8^, Isabelle Huys^1^

^1^Clinical Pharmacology and Pharmacotherapy, Department of Pharmaceutical and Pharmacological Sciences, KU Leuven, Leuven, Belgium

^2^Distributed and Secure Software, Department of Computer Science, KU Leuven, Leuven, Belgium

^3^KU Leuven Digital Society Institute, KU Leuven, Leuven, Belgium

^4^Centre for Biomedical Ethics and Law, Department of Public Health and Primary Care, KU Leuven, Leuven, Belgium

^5^Department of Gastroenterology and Hepatology, University Hospitals Leuven, KU Leuven, Leuven, Belgium

^6^Leuven University Vaccinology Centre, Department of Public Health and Primary Care, KU Leuven, Leuven, Belgium

^7^Department of Endocrinology, University Hospitals Leuven, KU Leuven, Leuven, Belgium

^8^Clinical and Experimental Endocrinology, Department of Chronic Diseases and Metabolism, KU Leuven, Leuven, Belgium

**Additional file 1: Visualization of provotype 5: contacting the principal investigator during the course of the study**


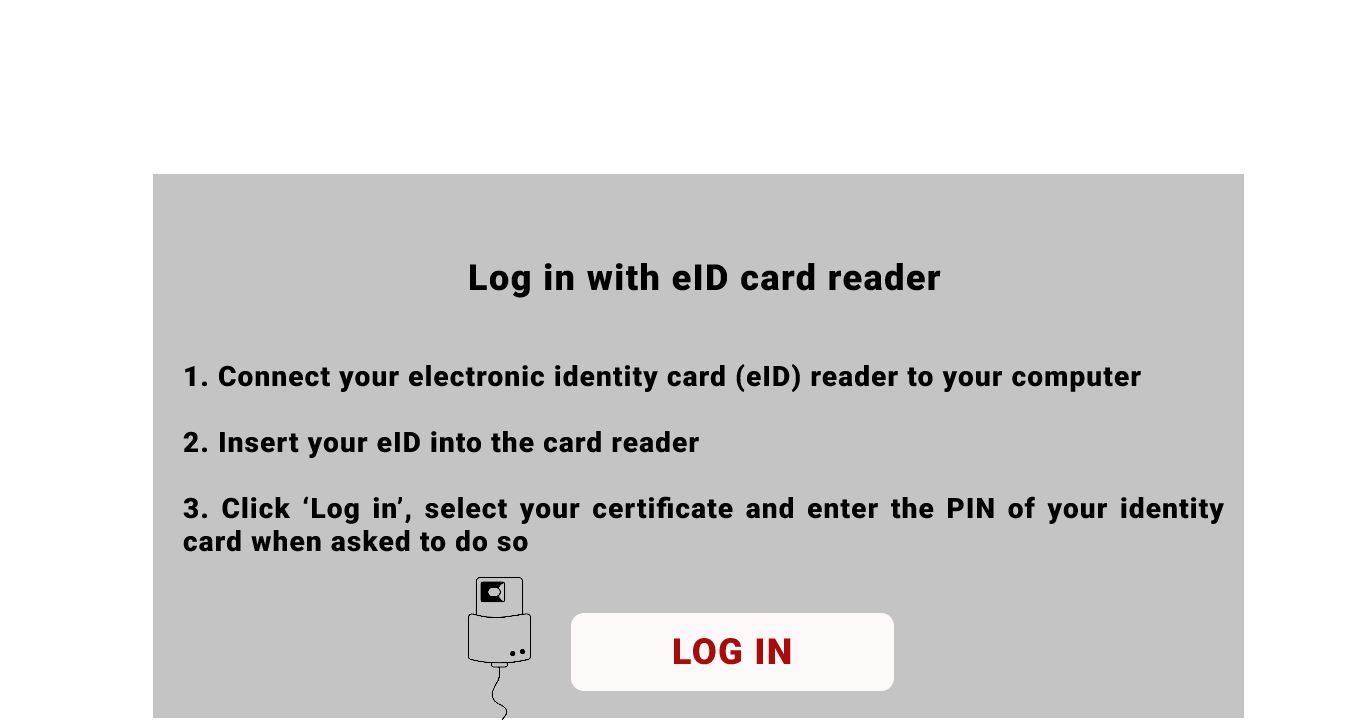


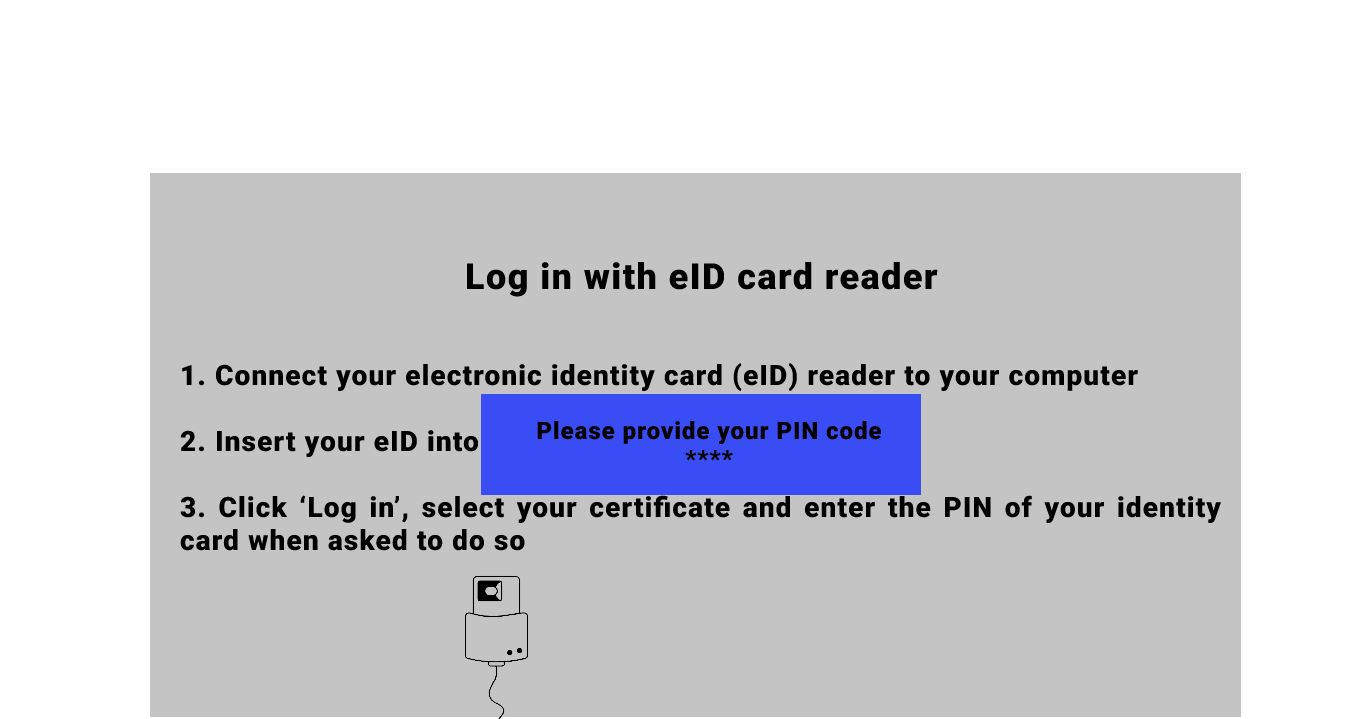


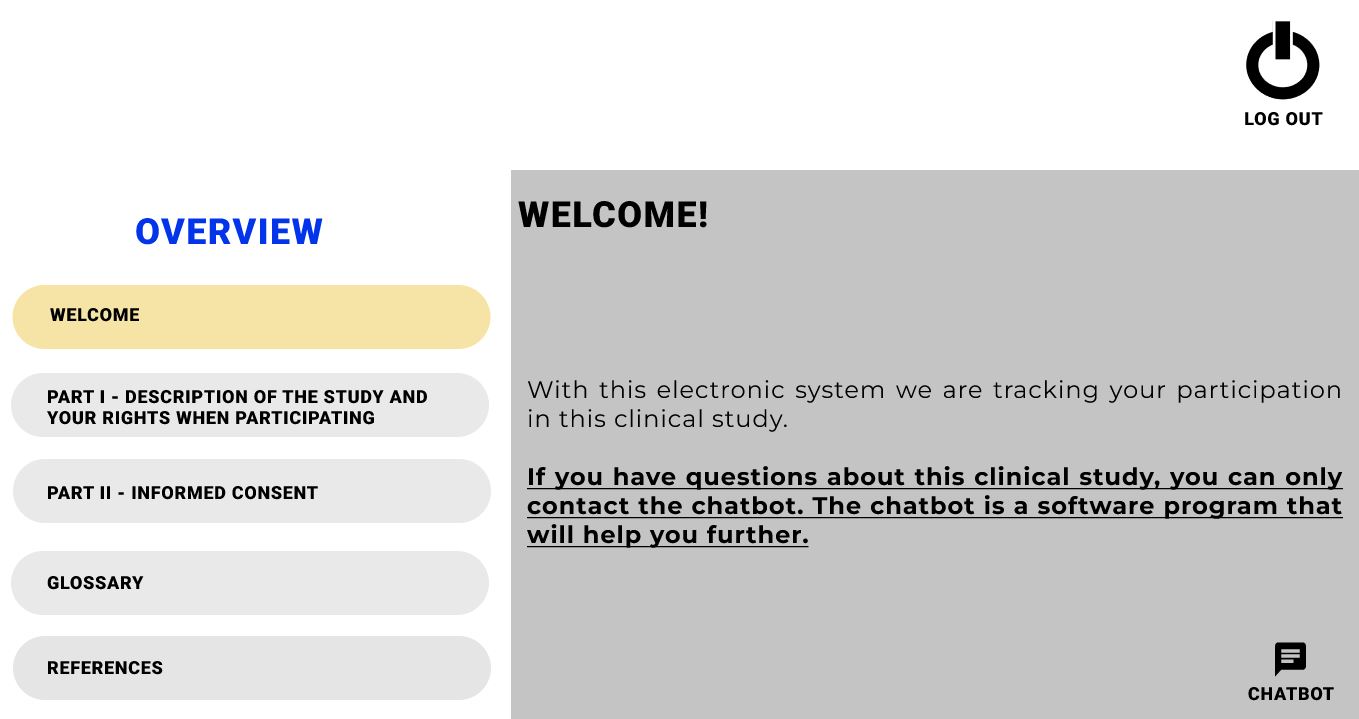


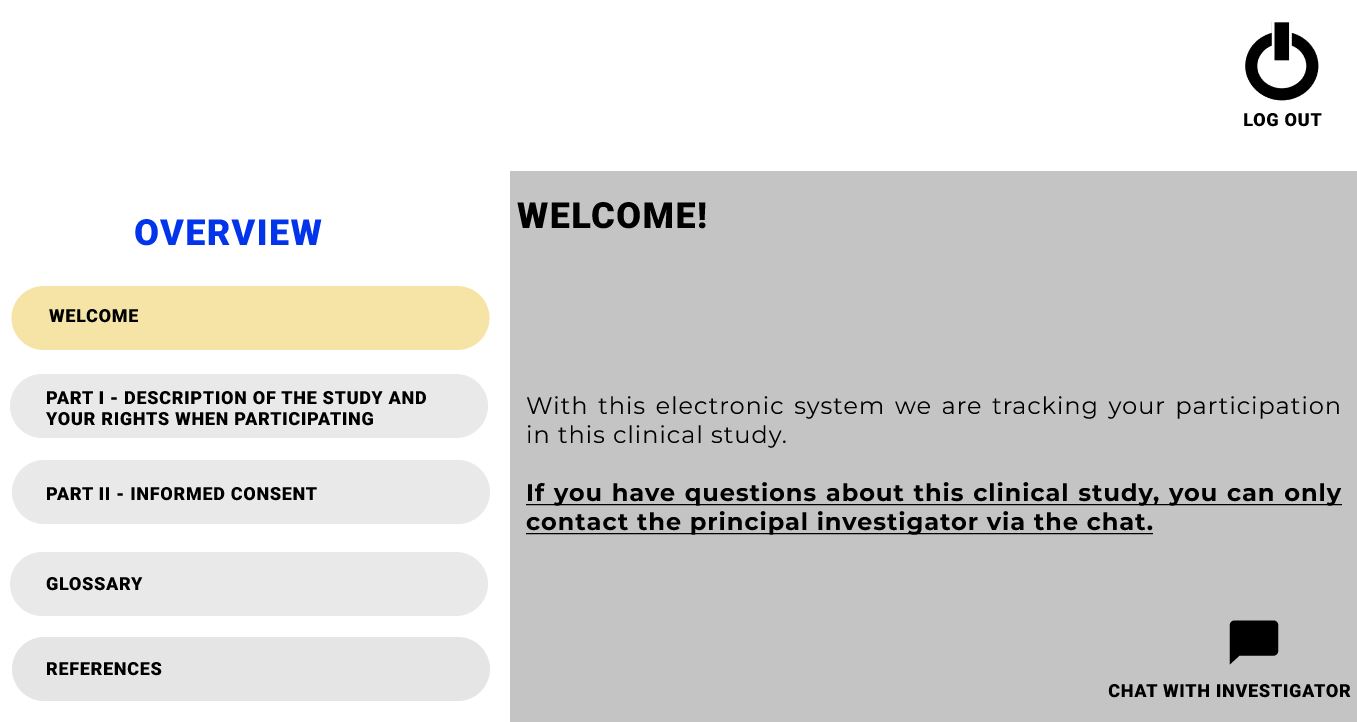


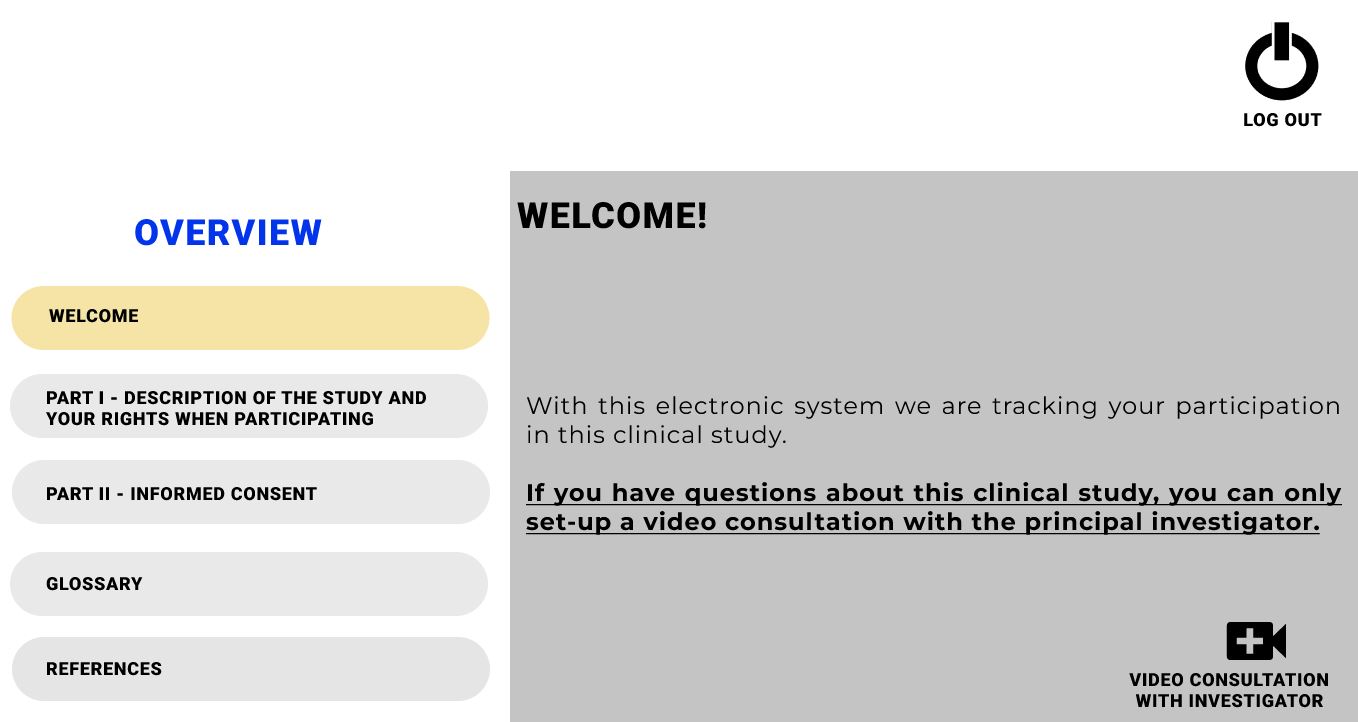


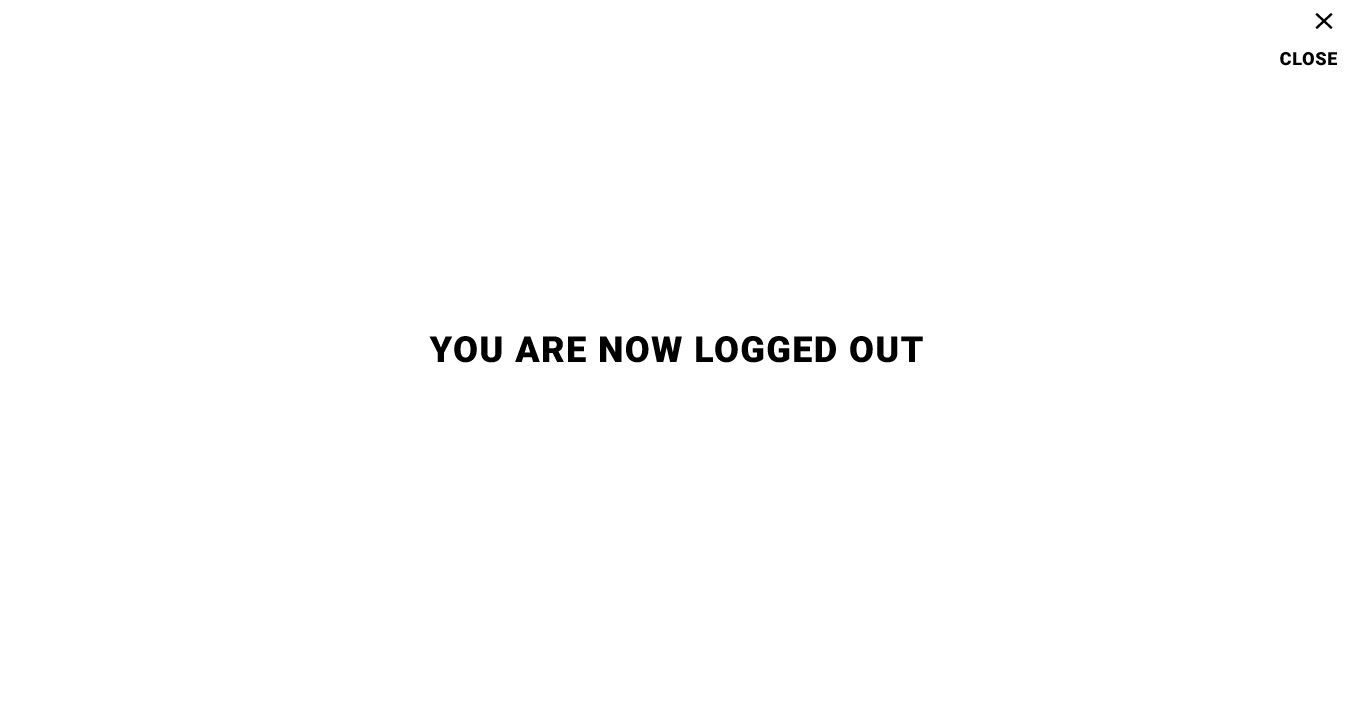

Supplement: Supplementary file 1 — Additional file 1: Visualization of provotype 5: contacting the principal investigator during the course of the study. [file 12911_2022_2039_MOESM1_ESM.docx]
