# Supplementary material for: Using provocative design to foster electronic informed consent innovation
Source: BMC Med Inform Decis Mak. 2022 Nov 17;22:296. doi: 10.1186/s12911-022-02039-6 (PMC9669523; doi:10.1186/s12911-022-02039-6)
Supplement: Supplementary file 2 — Additional file 2: Coding tree. [file 12911_2022_2039_MOESM2_ESM.docx]

**Using provocative design to foster electronic informed consent innovation**

Evelien De Sutter^1^, Stef Verreydt^2^, Koen Yskout^2^, David Geerts^3^, Pascal Borry^4^, An Outtier^5^, Marc Ferrante^5^, Corinne Vandermeulen^6^, Nele Vanmechelen^7^, Bart Van der Schueren^7,8^, Isabelle Huys^1^

^1^Clinical Pharmacology and Pharmacotherapy, Department of Pharmaceutical and Pharmacological Sciences, KU Leuven, Leuven, Belgium

^2^Distributed and Secure Software, Department of Computer Science, KU Leuven, Leuven, Belgium

^3^KU Leuven Digital Society Institute, KU Leuven, Leuven, Belgium

^4^Centre for Biomedical Ethics and Law, Department of Public Health and Primary Care, KU Leuven, Leuven, Belgium

^5^Department of Gastroenterology and Hepatology, University Hospitals Leuven, KU Leuven, Leuven, Belgium

^6^Leuven University Vaccinology Centre, Department of Public Health and Primary Care, KU Leuven, Leuven, Belgium

^7^Department of Endocrinology, University Hospitals Leuven, KU Leuven, Leuven, Belgium

^8^Clinical and Experimental Endocrinology, Department of Chronic Diseases and Metabolism, KU Leuven, Leuven, Belgium

## Additional file 2: Coding tree

| **Code** | **Sub-code level 1** | **Sub-code level 2** | **Description** |
| --- | --- | --- | --- |
| Hosting | Pharmaceutical company |  | Who is best placed to manage an eIC system? |
|  | Belgian government |  |  |
|  | Hospital |  |  |
|  | Principal investigator |  |  |
|  | Preferences |  |  |
|  | Advantages |  |  |
| Level of detail on access information | Not possible to provide information |  | How much information must be provided about access of stakeholders to an eIC system? |
|  | Legitimate rights |  |  |
|  | Detailed information |  |  |
|  | Minimal information |  |  |
| Being searchable | Physicians all over the world |  | What are the participant’s views on being searchable for future research studies? |
|  | Pharmaceutical company |  |  |
|  | Hospital |  |  |
|  | Type of studies |  |  |
| Being alerted about new information | Method of being alerted |  | How does the participant would like to be informed about new information? |
|  | Opinion on being alerted |  |  |
| Contact with the principal investigator | Chatbot |  | What is the opinion of the participant on methods to contact the principal investigator? |
|  | Chatbox |  |  |
|  | Video consultation |  |  |
|  | Personal contact |  |  |
| Reconsenting | Automatically agreeing |  | What are the most optimal conditions when asking the participant’s reconsent? |
|  | Highlighting changes |  |  |
|  | Additional information changes |  |  |
|  | Opinion on signature |  |  |
| Responsibility | Opinion on responsibility |  | How does the participant experience the responsibility when indicating which types of information he or she would like to receive? |
|  | Opinion on options | Status |  |
|  |  | Preliminary results |  |
|  |  | Final results |  |
|  |  | Additional investigations |  |
|  |  | New informed consent version |  |
| Test | Single attempt |  | How can a test be effectively integrated in an eIC system? |
|  | Multiple attempts |  |  |
|  | Additional explanation |  |  |
|  | Skipping questions - incorrect questions |  |  |
|  | Opinion on question and answer session |  |  |
| Age |  |  | How old is the participant? |
| Education |  |  | What is the highest education level of the participant? |
